# Dynamic regulation of genetic pathways and targets during aging in *Caenorhabditis elegans*

**DOI:** 10.18632/aging.100648

**Published:** 2014-03-31

**Authors:** Kan He, Tao Zhou, Jiaofang Shao, Xiaoliang Ren, Zhongying Zhao, Dahai Liu

**Affiliations:** ^1^Center for Stem Cell and Translational Medicine, School of Life Sciences, Anhui University, Hefei City, Anhui, P. R. China, 230601; ^2^Department of Biology, Hong Kong Baptist University, Hong Kong, China

**Keywords:** aging, C. elegans, pathways, network

## Abstract

Numerous genetic targets and some individual pathways associated with aging have been identified using the worm model. However, less is known about the genetic mechanisms of aging in genome wide, particularly at the level of multiple pathways as well as the regulatory networks during aging. Here, we employed the gene expression datasets of three time points during aging in *Caenorhabditis elegans* (*C. elegans*) and performed the approach of gene set enrichment analysis (GSEA) on each dataset between adjacent stages. As a result, multiple genetic pathways and targets were identified as significantly down- or up-regulated. Among them, 5 truly aging-dependent signaling pathways including MAPK signaling pathway, mTOR signaling pathway, Wnt signaling pathway, TGF-beta signaling pathway and ErbB signaling pathway as well as 12 significantly associated genes were identified with dynamic expression pattern during aging. On the other hand, the continued declines in the regulation of several metabolic pathways have been demonstrated to display age-related changes. Furthermore, the reconstructed regulatory networks based on three of aging related Chromatin immunoprecipitation experiments followed by sequencing (ChIP–seq) datasets and the expression matrices of 154 involved genes in above signaling pathways provide new insights into aging at the multiple pathways level. The combination of multiple genetic pathways and targets needs to be taken into consideration in future studies of aging, in which the dynamic regulation would be uncovered.

## INTRODUCTION

The nematode worm *C. elegans* had already been established as a useful model organism for studies of aging since it has a relatively short life span and can easily propagate populations of synchronized individuals. Aging in *C. elegans* has been proposed to be caused by hyperfunction, that is, overactivity during adulthood of processes particularly biosynthetic that contribute to development and reproduction. Such hyperfunction can lead to hypertrophy-associated pathologies, which cause the age increase in death [1]. Several mutated genes have been identified to extend lifespan by large-scale RNAi longevity screens in *C. elegans* [2]. However, there are discordant results in some of the screens which suggest that these screens have not been saturated and more single gene mutations and deletions that influence lifespan and healthspan are conceivable [3]. Together, some signaling pathways defined by each genetic factors which affect lifespan have been discovered, such as insulin/IGF-1-like signaling (IIS) pathway by a FOXO-family transcription factor DAF-16 [4]. Undoubtedly, aging involves changes in multiple genes involved in multiple processes, although some of them have not yet been known. Currently, microarray technology has been widely used in the genome wide study of gene expression changes associated with aging in *C. elegans* and may provide some insights into potential mechanisms [5]. A lot of genes with specific changes related to *C. elegans* aging have been uncovered from the statistical analysis of differentially expressed genes (DEG) in the expression profiling, including the methods of Analysis of Variance (ANOVA), Significance Analysis of Microarray (SAM) and Student t test [5-8]. However many questions remain, especially on the regulation of genetic pathways and the expression patterns as well as regulatory networks during aging. Compared to individual DEG approach, the well-established approach of Gene Set Enrichment Analysis (GSEA) may be more compatible in the interpretation of gene expression data [9, 10].

As we all know, DNA-binding proteins, especially the transcription factors (TFs), play crucial roles in many major cellular processes. Nowadays, ChIP–seq can successfully detect the binding sites of TFs in genome wide [11, 12]. From the modENCODE database, the relevant ChIP–seq data sets involved in several aging transcriptional regulators are available [13]. In order to capture the essential regulatory features behind high-throughput biological data, such as identifying potential regulatory interactions between TFs and genes involved in aging, especially in multiple pathways level, it would be necessary and possible to integrate ChIP–seq data and gene expression profiles.

## RESULTS AND DISCUSSION

### Identification of significantly aging-dependent pathways

**Figure 1:**
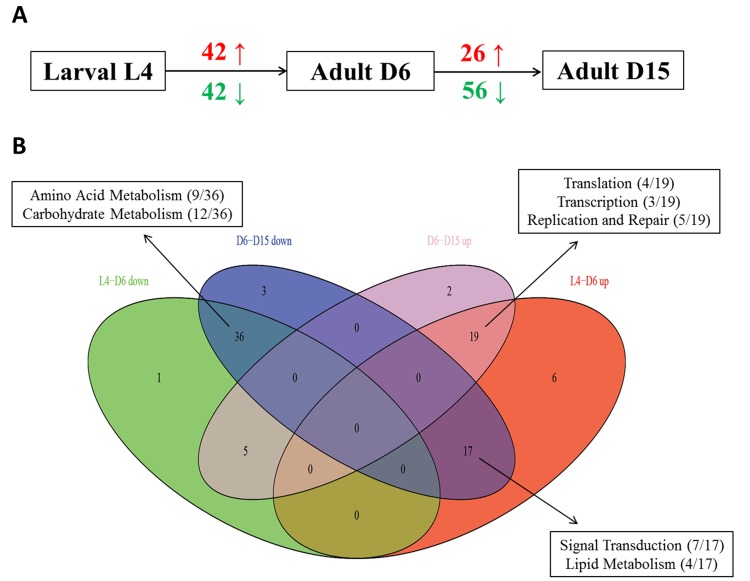
The summary of up- and down- regulated pathways based on GSEA during aging (A) It showed the numbers of significantly identified pathways based on GSEA in each period during aging in *C. elegans* (red is for up-regulated and green is for down-regulated). Obviously, there were 42 up-regulated and 42 down-regulated significantly associated pathways from the stage of L4 to D6, as well as 26 up-regulated and 56 down-regulated significantly associated pathways from the stage of D6 to D15. (B) The Venn diagram showed the comparisons of each pathway group above. L4-D6 down represents the group of identified down-regulated pathways from the stage of L4 to D6; L4-D6 up represents the group of identified up-regulated pathways from the stage of L4 to D6; D6-D15 down represents the group of identified down-regulated pathways from the stage of D6 to D15; D6-D15 up represents the group of identified up-regulated pathways from the stage of D6 to D15. The boxes showed the mainly involved functional categories (KEGG Pathway Maps) as well as the percentage of each category.

Based on the GSEA approach, we have identified several significantly related genetic pathways during aging in *C. elegans*. There were significantly 42 up- and 42 down-regulated pathways from the stage of L4 to D6, as well as 26 up- and 56 down-regulated pathways from the stage of D6 to D15 (Figure 1A and Additional file 1). By the comparison of each group, we have also identified some overlapping pathways significantly associated in both of these two periods, which were detailed in Table 1 and Figure 1B. In summary, there were totally 17 overlapping pathways significantly up-regulated from L4 to D6 but down-regulated from D6 to D15, and most of them were related to Signal Transduction (7/17) and Lipid Metabolism (4/17) according to KEGG pathway maps in the KEGG database (http://www.genome.jp/kegg/). Interestingly, 6 different signaling pathways were significantly par-ticipating in this dynamic regulation, including MAPK signaling pathway, mTOR signaling pathway, Wnt signaling pathway, TGF-beta signaling pathway, ErbB signaling pathway and Hedgehog signaling pathway.

**Table 1:** The overlapping significantly down or up-regulated pathways during aging

Overlapping Significant Pathways	KEGG Pathway Maps
**L4-D6 up / D6-D15 down**	
00562 Inositol phosphate metabolism	Carbohydrate Metabolism
04710 Circadian rhythm - mammal	Environmental Adaptation
00511 Other glycan degradation	Glycan Biosynthesis and Metabolism
04650 Natural killer cell mediated cytotoxicity	Immune System
00564 Glycerophospholipid metabolism	Lipid Metabolism
00590 Arachidonic acid metabolism	Lipid Metabolism
00600 Sphingolipid metabolism	Lipid Metabolism
00561 Glycerolipid metabolism	Lipid Metabolism
00450 Selenocompound metabolism	Metabolism of Other Amino Acids
04310 Wnt signaling pathway	Signal Transduction
04010 MAPK signaling pathway	Signal Transduction
04012 ErbB signaling pathway	Signal Transduction
**04350 TGF-beta signaling pathway**	**Signal Transduction**
04070 Phosphatidylinositol signaling system	Signal Transduction
**04150 mTOR signaling pathway**	**Signal Transduction**
**04340 Hedgehog signaling pathway**	**Signal Transduction**
04144 Endocytosis	Transport and Catabolism
**L4-D6 down / D6-D15 down**	
00280 Valine, leucine and isoleucine degradation	Amino Acid Metabolism
00310 Lysine degradation	Amino Acid Metabolism
00330 Arginine and proline metabolism	Amino Acid Metabolism
00250 Alanine, aspartate and glutamate metabolism	Amino Acid Metabolism
00270 Cysteine and methionine metabolism	Amino Acid Metabolism
00350 Tyrosine metabolism	Amino Acid Metabolism
00260 Glycine, serine and threonine metabolism	Amino Acid Metabolism
00380 Tryptophan metabolism	Amino Acid Metabolism
00340 Histidine metabolism	Amino Acid Metabolism
00650 Butanoate metabolism	Carbohydrate Metabolism
00052 Galactose metabolism	Carbohydrate Metabolism
00040 Pentose and glucuronate interconversions	Carbohydrate Metabolism
00010 Glycolysis / Gluconeogenesis	Carbohydrate Metabolism
00051 Fructose and mannose metabolism	Carbohydrate Metabolism
00640 Propanoate metabolism	Carbohydrate Metabolism
00500 Starch and sucrose metabolism	Carbohydrate Metabolism
00020 Citrate cycle (TCA cycle)	Carbohydrate Metabolism
00630 Glyoxylate and dicarboxylate metabolism	Carbohydrate Metabolism
00620 Pyruvate metabolism	Carbohydrate Metabolism
00030 Pentose phosphate pathway	Carbohydrate Metabolism
00053 Ascorbate and aldarate metabolism	Carbohydrate Metabolism
00514 Other types of O-glycan biosynthesis	Glycan Biosynthesis and Metabolism
00062 Fatty acid elongation	Lipid metabolism
00910 Nitrogen metabolism	Lipid metabolism
00071 Fatty acid metabolism	Lipid Metabolism
01100 Metabolic pathways	Metabolism
00860 Porphyrin and chlorophyll metabolism	Metabolism of Cofactors and Vitamins
00670 One carbon pool by folate	Metabolism of Cofactors and Vitamins
00830 Retinol metabolism	Metabolism of Cofactors and Vitamins
00480 Glutathione metabolism	Metabolism of Other Amino Acids
00410 beta-Alanine metabolism	Metabolism of Other Amino Acids
04020 Calcium signaling pathway	Signal Transduction
04145 Phagosome	Transport and Catabolism
00983 Drug metabolism - other enzymes	Xenobiotics Biodegradation and Metabolism
00980 Metabolism of xenobiotics by cytochrome P450	Xenobiotics Biodegradation and Metabolism
00982 Drug metabolism - cytochrome P450	Xenobiotics Biodegradation and Metabolism
**L4-D6 up / D6-D15 up**	
04914 Progesterone-mediated oocyte maturation	Endocrine System
04120 Ubiquitin mediated proteolysis	Folding, Sorting and Degradation
03018 RNA degradation	Folding, Sorting and Degradation
00510 N-Glycan biosynthesis	Glycan Biosynthesis and Metabolism
00240 Pyrimidine metabolism	Nucleotide Metabolism
03420 Nucleotide excision repair	Replication and Repair
03030 DNA replication	Replication and Repair
03430 Mismatch repair	Replication and Repair
03410 Base excision repair	Replication and Repair
03440 Homologous recombination	Replication and Repair
04630 Jak-STAT signaling pathway	Signal Transduction
03022 Basal transcription factors	Transcription
03020 RNA polymerase	Transcription
03040 Spliceosome	Transcription
03010 Ribosome	Translation
03013 RNA transport	Translation
03015 mRNA surveillance pathway	Translation
03008 Ribosome biogenesis in eukaryotes	Translation
04140 Regulation of autophagy	Transport and Catabolism
**L4-D6 down / D6-D15 up**	
03060 Protein export	Folding, Sorting and Degradation
00230 Purine metabolism	Nucleotide Metabolism
00970 Aminoacyl-tRNA biosynthesis	Translation
00360 Phenylalanine metabolism	Amino Acid Metabolism
00290 Valine, leucine and isoleucine biosynthesis	Amino Acid Metabolism

Moreover, there were totally 36 overlapping pathways significantly down-regulated both from L4 to D6 and from D6 to D15, and most of them were related to Amino Acid Metabolism (9/36) and Carbohydrate Metabolism (12/36); there were totally 19 overlapping pathways significantly up-regulated both from L4 to D6 and from D6 to D15, and most of them were related to Translation (4/19) and Transcription (3/19) as well as Replication and Repair (5/19). All of these data revealed the dynamic re-gulation in multiple pathways during aging in *C. elegans*.

### Dynamic regulation in multiple signaling pathways

**Figure 2:**
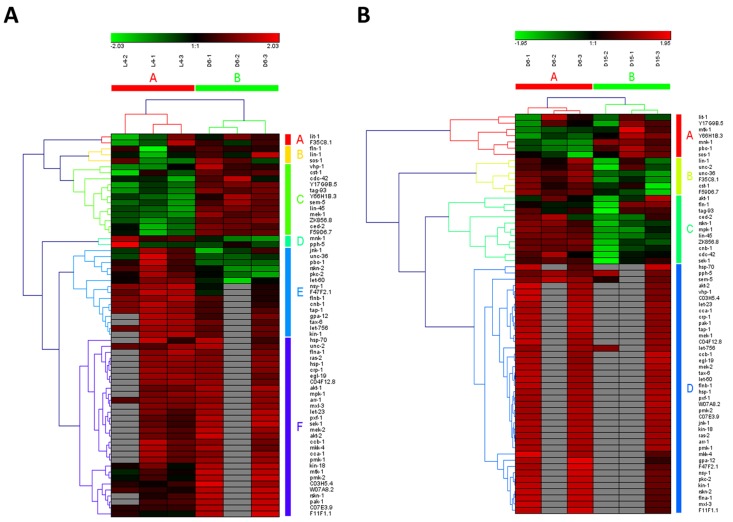
The heat map and hierarchical clustering in MAPK signaling pathway during aging (**A**) It showed the heat map and hierarchical clustering in MAPK signaling pathway from the stage of L4 to D6. The samples (column) were clustered into two groups, three replicates in the stage of L4 (L4-1, L4-2 and L4-3) were clustered together and three replicates at day 6 (D6-1, D6-2 and D6-3) were clustered together. There were 64 involved genes in MAPK signaling pathway from L4 to D6, which were clustered into 6 groups (the group from A to F).(**B**) It showed the heat map and hierarchical clustering in MAPK signaling pathway from the stage of D6 to D15. The samples were clustered into two groups, three replicates at day 6 (D6-1, D6-2 and D6-3) were clustered together and three replicates at day 15 (D15-1, D15-2 and D15-3) were clustered together. There were also 64 involved genes in MAPK signaling pathway from D6 to D15, which were clustered into 4 groups (the group from A to D). Red is for up-regulated and green is for down-regulated.

In the study of Youngman MJ et al. [14], a decline in PMK-1 p38 mitogen-activated protein kinase (MAPK) pathway, one of conserved pathways involved in pathogen defense, was observed by analyzing gene expression levels using unpaired t test in synchronized populations of N2 worms during mid-to-late adulthood (at Day 6 of adulthood and Day 15 of adulthood). It was consistent with our result on MAPK signaling pathway, which was identified as significantly down-regulated from D6 to D15 of adulthood (highlighted in red color in Additional file 1). Surprisingly, MAPK signaling pathway was shown to be significantly up-regulated during young-to-mid adulthood (at the late larval L4 stage and Day 6 of adulthood) in our study, which was not reported by Youngman MJ et al. study [14]. Furthermore, MAPK signaling pathway was identified as truly aging-dependent pathways based on the hierarchical clustering of both samples and involved genes (Figure 2A for the data from L4 to D6 and Fig2B from D6 to D15). Obviously, using the expression data of involved genes in MAPK signaling pathway, six samples were clustered into two groups with samples at the same stage (L4-1, L4-2 and L4-3 in group of L4; D6-1, D6-1 and D6-3 in group of D6; D15-1, D15-1 and D15-3 in group of D15). Actually, MAP kinase (MAPK)/Receptor Tyrosine Kinase (RTK)/RasGTPase signaling pathways were used repeatedly during metazoan development, which were reported to be required for larval viability and for many different developmental processes, including induction of vulval, uterine, spicule, P12 and excretory duct cell fates, control of sex myoblast migration and axon guidance, and promotion of germline meiosis [15]. The core components or regulators of *C. elegans* RTK/Ras/MAPK signaling pathway were also identified as the aging-dependent targets [16-18], such as LET-23, LIN-1 and LIN-45 (Figure 2 and Additional file 2). Among these candidate targets, four interesting genes, including *ced-2*, *lin-45*, *ZK856.8* and *F59D6.7*, were significantly up-regulated from L4 to D6 and down-regulated from D6 to D15, showing dynamic expression patterns. Their significances of gene expression changes between two adjacent stages were shown in Table 2. In *C. elegans*, the *ced-2* gene encodes a Src homology (SH) 2 and 3-containing adaptor protein, homologous to human CrkII, which is required for phagocytosis during programmed cell death and for migration of the distal tip cells of the somatic gonad. It was previously reported that *ced-2* mutants may exhibit persistant corpses indicating defects in phagocytosis and distal tip cell migration defects in the gonadal arms [19]. The *lin-45* gene encodes an ortholog of the vertebrate protein RAF which is required for larval viability, fertility and the induction of vulval cell fates [18]. Based on RNAi studies of three genes constituting the ERK cascade (*lin-45*/RAF1, *mek-2*/MEK1/2, and*mpk-1*/ERK1/2) as well as *lip-1* encoding a MAPK phosphatase that inactivates MPK-1, a novel ERK-MAPK-mediated signaling pathway has been identified, which promotes longevity through two candidate transcription factors, SKN-1 and DAF-16 [20]. Additionally, the genes of *ZK856.8* and *F59D6.7* would be two important targets for further study of aging in MAPK signaling pathway.

**Table 2:** The significantly associated genes with dynamic regulation pattern in signaling pathways during aging

Genes	Related pathways	p1 (L4 to D6)	p2 (D6 to D15)
*ced-2*	MAPK signaling pathway	1.70E-02	3.62E-04
*lin-45*	MAPK signaling pathway mTOR signaling pathway ErbB signaling pathway	1.34E-03	1.87E-03
*ZK856.8*	MAPK signaling pathway	1.03E-03	7.11E-03
*F59D6.7*	MAPK signaling pathway	1.41E-02	2.17E-02
*pdk-1*	mTOR signaling pathway	4.58E-04	1.19E-02
*cyd-1*	Wnt signaling pathway	4.50E-03	1.58E-03
*sma-4*	Wnt signaling pathway TGF-beta signaling pathway	9.60E-03	5.51E-03
*skr-7*	Wnt signaling pathway TGF-beta signaling pathway	3.76E-06	2.05E-02
*skr-8*	TGF-beta signaling pathway	3.12E-05	4.59E-02
*skr-9*	Wnt signaling pathway TGF-beta signaling pathway	1.03E-04	3.57E-02
*skr-13*	Wnt signaling pathway TGF-beta signaling pathway	1.34E-05	2.27E-03
*skr-14*	Wnt signaling pathway TGF-beta signaling pathway	5.99E-05	6.63E-03

**Figure 3:**
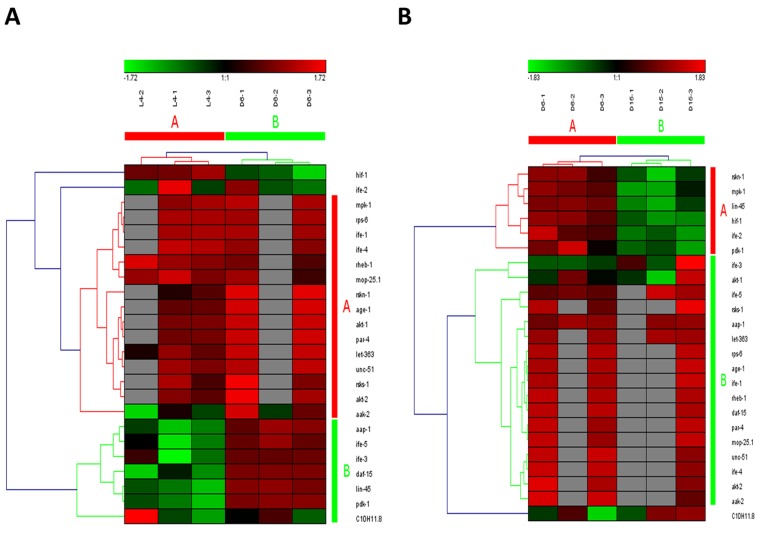
The heat map and hierarchical clustering in mTOR signaling pathway during aging (**A**) It showed the heat map and hierarchical clustering in mTOR signaling pathway from the stage of L4 to D6. The samples (column) were clustered into two groups, three replicates in the stage of L4 (L4-1, L4-2 and L4-3) were clustered together and three replicates at day 6 (D6-1, D6-2 and D6-3) were clustered together. There were 24 involved genes (row) in mTOR signaling pathway from L4 to D6, which were clustered into 2 groups (the group of A and B). (**B**) It showed the heat map and hierarchical clustering in mTOR signaling pathway from the stage of D6 to D15. The samples were clustered into two groups, three replicates at day 6 (D6-1, D6-2 and D6-3) were clustered together and three replicates at day 15 (D15-1, D15-2 and D15-3) were clustered together. There were also 24 involved genes in mTOR signaling pathway from D6 to D15, which were clustered into 2 groups (the group of A and B).Red is for up-regulated and green is for down-regulated.

The mammalian target of rapamycin (mTOR), also known as mechanistic target of rapamycin has been identified as a key modulator of ageing and age-related disease. The fact that inhibition of mTOR signaling pathway may extend lifespan in model organisms and confer protection against a growing list of age-related pathologies has been clearly approved [21]. In our study, this pathway was also identified as one of truly aging-dependent pathways with dynamic regulation, which was significantly up-regulated from L4 to D6 and down-regulated from D6 to D15 (Table 1). Like MAPK signaling pathway, based on the expression of candidate genes in mTOR signaling pathway, the samples at the same time point can be clustered together (Figure 3A for the data from L4 to D6 and Figure 3B from D6 to D15). There were 24 genes involved in mTOR signaling pathway both in the period of young-to-mid and mid-to- late adulthood, which were both clustered into 2 groups (the group of A and B in Figure 3A & 3B). Among these genes appearing in both the group of B in Figure 3A and the group A in Figure 3B, besides *lin-45* mentioned above, the gene of *pdk-1* was identified with dynamic expression pattern, which was significantly up-regulated from L4 to D6 (p=4.58E-04) and down-regulated from D6 to D15 (p=1.19E-02) (Table 2).In *C. elegans*, *pdk-1* encodes the 3-phosphoinositide-dependent kinase 1 ortholog (PDK-1), which is a component of the DAF-2/insulin receptor-like signaling pathway and accordingly, functions to regulate such processes as dauer larvae formation as well as longevity [21]. The increased lifespan in *daf-2* insulin/IGF-1 receptor mutants is accompanied by up-regulation of the MDL-1 Mad basic helix-loop-helix leucine zipper transcriptionfactor, which is an inhibitor of cell proliferation and growth that slows progression of an age-related pathology in *C. elegans* [22]. The previous studies have illustrated that mTOR signaling pathway plays the central role, whereas other pathways act on it by activating or antagonizing either at upstream or downstream [23-26].

**Figure 4:**
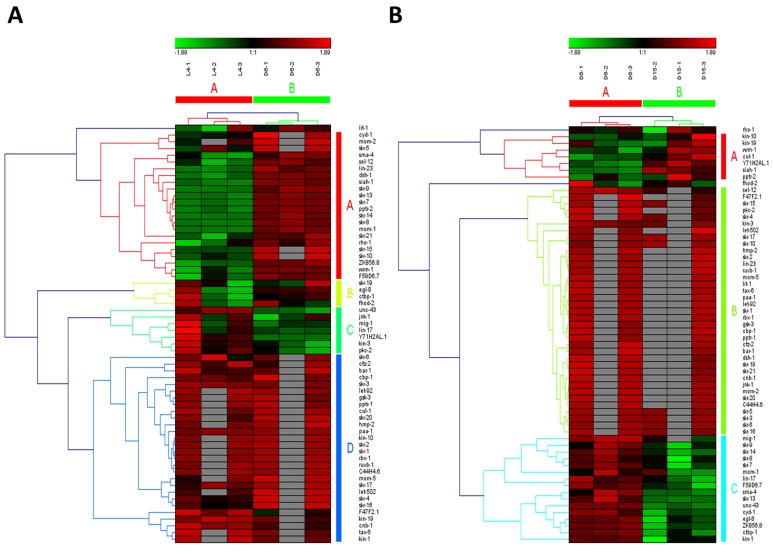
The heat map and hierarchical clustering in Wnt signaling pathway during aging (**A**) It showed the heat map and hierarchical clustering in Wnt signaling pathway from the stage of L4 to D6. The samples (column) were clustered into two groups, three replicates in the stage of L4 (L4-1, L4-2 and L4-3) were clustered together and three replicates at day 6 (D6-1, D6-2 and D6-3) were clustered together. There were 62 involved genes (row) in Wnt signaling pathway from L4 to D6, which were clustered into 4 groups (the group from A to D). (**B**) It showed the heat map and hierarchical clustering in Wnt signaling pathway from the stage of D6 to D15. The samples were clustered into two groups, three replicates at day 6 (D6-1, D6-2 and D6-3) were clustered together and three replicates at day 15 (D15-1, D15-2 and D15-3) were clustered together. There were also 62 involved genes in Wnt signaling pathway from D6 to D15, which were clustered into 3 groups (the group from A to C). Red is for up-regulated and green is for down-regulated.

**Figure 5:**
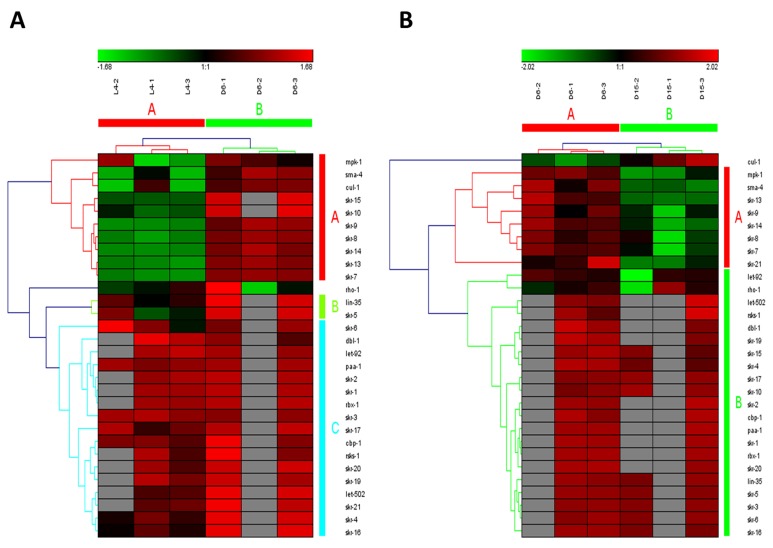
The heat map and hierarchical clustering in TGF-beta signaling pathway during aging (**A**) It showed the heat map and hierarchical clustering in TGF-beta signaling pathway from the stage of L4 to D6. The samples (column) were clustered into two groups, three replicates in the stage of L4 (L4-1, L4-2 and L4-3) were clustered together and three replicates at day 6 (D6-1, D6-2 and D6-3) were clustered together. There were 30 involved genes (row) in TGF-beta signaling pathway from L4 to D6, which were mainly clustered into 3 groups (the group from A to C). (**B**) It showed the heat map and hierarchical clustering in TGF-beta signaling pathway from the stage of D6 to D15. The samples were clustered into two groups, three replicates at day 6 (D6-1, D6-2 and D6-3) were clustered together and three replicates at day 15 (D15-1, D15-2 and D15-3) were clustered together. There were also 30 involved genes in TGF-beta signaling pathway from D6 to D15, which were mainly clustered into 2 groups (the group of A and B).Red is for up-regulated and green is for down-regulated.

Wnt signaling pathways have extremely diverse functions in animals, including the control of gene expression, cell behavior, cell adhesion, and cell polarity [19]. During aging in *C. elegans*, Wnt signaling pathway was identified as another truly aging-dependent pathway with dynamic regulation, which was significantly up-regulated from L4 to D6 and down-regulated from D6 to D15. There were 62 involved genes in Wnt signaling pathway, which were clustered into 4 groups from L4 to D6 (the group from A to D in Figure 4A) and clustered into 3 groups (the group from A to C in Figure 4B). Among these genes appearing in both the group of A in Figure 4A and the group C in Figure 4B, there were 8 dynamically regulated genes containing *sma-4*, *cyd-1*, *skr-7,9,13,14*,*F59D6.7* and *ZK856.8*, which were significantly up-regulated from L4 to D6 and down- regulated from D6 to D15(Table 2).The gene of *sma-4* encodes a Smad protein and a homolog of human DPC4, the protein of SMA-4 is similar to members of the vertebrate protein family of Dwarfins. During development, *sma-4* functions as part of a DBL-1/SMA-6 TGF-beta-related signaling pathway that controls body size and male tail sensory ray and spicule formation as well as regulates reproductive aging via oocyte and germline quality maintenance [27]. Indeed, TGF-beta signaling pathway was one of our candidate aging-dependent pathways (Figure 5). The Smad-mediated TGF-beta signaling pathway controls numerous cellular responses from cell proliferation, differentiation and extracellular matrix remodelling to embryonic development in species ranging from worms to mammals [20]. It has been shown that TGF-beta superfamily ligands can regulate cellular or physiological processes through non-canonical pathways by the activation of other signaling molecules, e.g. Akt, MAPK, mTOR, and Src independent of Smad proteins [28]. In this study, Wnt signaling pathway would be seen as one of Smad-dependent pathways in TGF-beta family signaling based on the common regulator of SMA-4 (Table 2).

**Figure 6:**
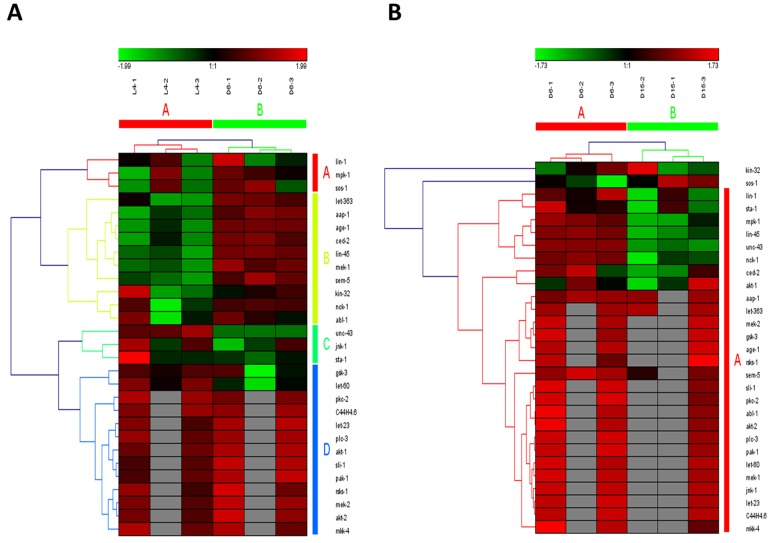
The heat map and hierarchical clustering in ErbB signaling pathway during aging (**A**) It showed the heat map and hierarchical clustering in ErbB signaling pathway from the stage of L4 to D6. The samples (column) were clustered into two groups, three replicates in the stage of L4 (L4-1, L4-2 and L4-3) were clustered together and three replicates at day 6 (D6-1, D6-2 and D6-3) were clustered together. There were 29 involved genes (row) in ErbB signaling pathway from L4 to D6, which were clustered into 4 groups (the group from A to D). (**B**) It showed the heat map and hierarchical clustering in ErbB signaling pathway from the stage of D6 to D15. The samples were clustered into two groups, three replicates at day 6 (D6-1, D6-2 and D6-3) were clustered together and three replicates at day 15 (D15-1, D15-2 and D15-3) were clustered together. There were also 29 involved genes in ErbB signaling pathway from D6 to D15, in which there was mainly one group (the group of A).Red is for up-regulated and green is for down-regulated.

ErbB signaling pathway was the last one of our truly aging-dependent signaling pathways with dynamic expression pattern during aging (Figure 6). The ErbB family of receptor tyrosine kinases (RTKs) couples binding of extracellular growth factor (EGF) ligands to intracellular signaling pathways regulating diverse biologic responses, including proliferation, differentiation, cell motility, and survival [29]. In this pathway, *lin-45* was the only one significantly regulated gene, which also appearing in both MAPK signaling pathway and mTOR signaling pathway (Table 2). *lin-45* is a component of an EGFR-mediated inductive signaling pathway that causes vulva precursor cells (VPCs) to generate the vulva [30]. Expression of LIN-45(V627E) and LIN-45(ED) was previously identified to occasionally cause the vulva to burst at the L4-to-adult molt, which may reflect defects in either the VPC specification or the resulting vulval cells [31]. Although Hedgehog signaling pathway has been identified as one of the significantly aging-dependent pathways, it was not the true one due to the unsuccessful clustering on samples from D6 to D15 by using involved genes (Additional file 3).

**Figure 7:**
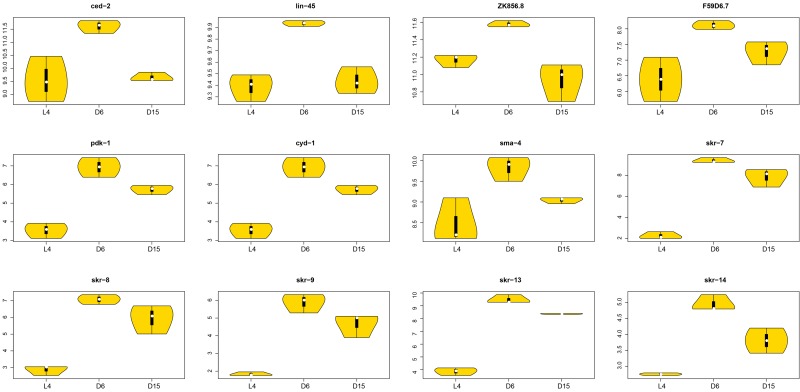
The violin plots of significantly associated genes in signaling pathways during aging The violin plots showed the expression distributions of the significantly associated genes with dynamic regulation pattern in signaling pathways during aging. The X-axis represents three different stages (L4, D6 and D15); The Y-axis represents the normalized gene expression value. There were 12 genes (*ced-2, lin-45, ZK856.8, F59D6.7, pdk-1, cyd-1, sma-4*, and *skr-7, 8, 9, 13, 14*), all of which were significantly up-regulated from L4 to D6 and down-regulated from D6 to D15. The significances of each gene were shown in Table 2.

Generally, there were 12 significant genes with dynamic regulation patterns during aging in above five truly significant signaling pathways (Figure 7). They were *ced-2, lin-45, ZK856.8, F59D6.7, pdk-1, cyd-1, sma-4,* and*skr-7, 8, 9, 13, 14*, all of which were significantly up-regulated from L4 to D6 and down-regulated from D6 to D15. The information of significances and involved pathways were shown in Table 2. These targets would be regarded as key factors in future studies of genetic mechanisms of aging. Interestingly, 5 members of *skr* gene family (*skr-7, 8, 9, 13, 14*) were included. *skr-7* encodes a homolog of Skp1 that functions within a particular SCF ubiquitin-ligase (E3) complex by binding both a cullin (a homolog of S. cerevisiae Cdc53) and an F box protein (through direct interaction with an F box motif), which is required for posterior body morphogenesis, embryonic and larval development, and cell proliferation [32, 33]. The most closely related paralogs of *skr-7, -8 and -9* were highly similar, but it is not clear whether these genes comprise a functionally redundant set. The function of SKR-13 is not necessarily confined to, or even partially involved with *C. elegans* ubiquitin-ligase complexes. In two-hybrid assays, SKR-13 does not bind to any known *C. elegans* cullins (CUL-1 through CUL-6) [33]. The most closely related paralogs of *skr-13* in the *C. elegans* genome are *skr-12* and *skr-14.*

### Continued decline in metabolism related pathways

Besides signal transduction, several measurements of metabolic activity have been demonstrated to display age-related changes. For example, about 50% of declines in carbon dioxide generation by populations of live worms grown on plates measured by gas respirometry have been found from day 6 to day 12 [34]. Additionally, about 60% of declines in oxygen consumption of populations of live worms in liquid culture monitored polarographically using Clark type electrodes have also been evaluated from adult day 0 to day 12 [35, 36]. By contrast, it was indicated that a reduced metabolic rate may cause the extended lifespan of worms based on the result of reduced metabolic rate of *age-1* and *daf-2* mutants compared to wild-type animals [34]. In our study, most of metabolism related pathways were significantly identified with continued decline in expression pattern both from L4 to D6 and from D6 to D15.It has been reported that the couplingof a metabolic shift with endotoxin detoxification results in extreme longevity following *mekk-3* knock-down, a mammalian MEKK3-like kinase gene in *C. elegans* [37]. An age-dependent decreased abundance of proteins involved in mitochondrial function, electron transport chain, citric acid cycle, and fatty acid metabolism as well as increased abundance of proteins involved in glycolysis and oxidative stress response have been revealed by the quantitative proteomics and pathway analysis [38].

### Multi-level gene regulatory networks in signaling pathways during aging

**Figure 8:**
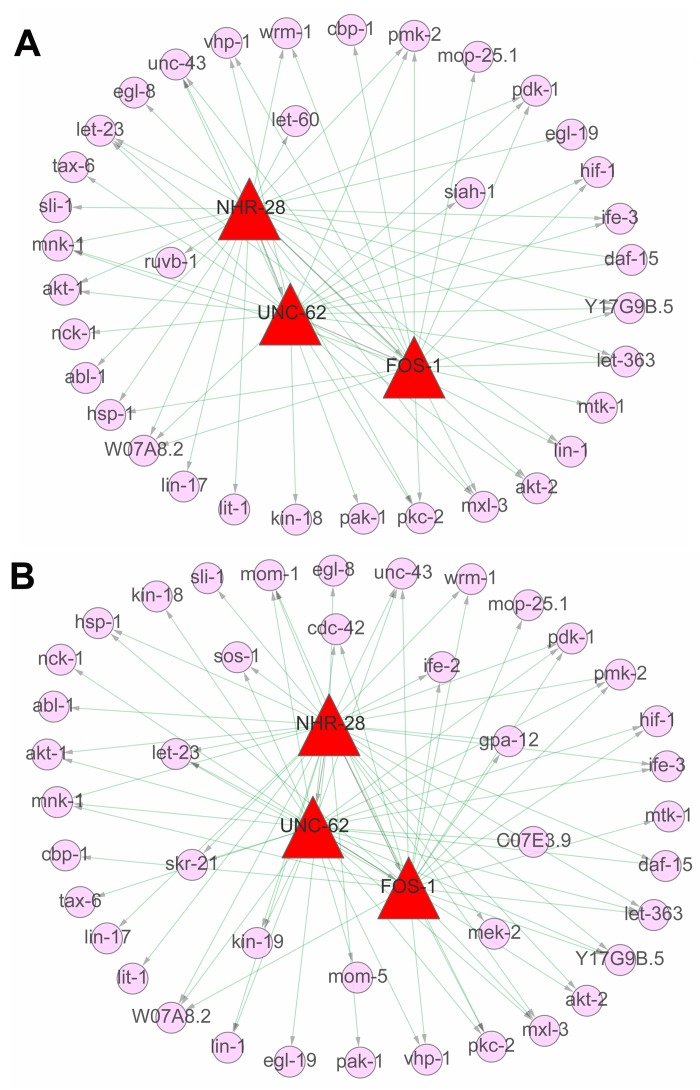
The regulatory networks in signaling pathways during aging The regulatory networks were constructed based on three of aging related ChIP–seq datasets (3 TFs of NHR-28, UNC-62 and FOS-1 related) and the expression matrices of 154 involved genes in identified signaling pathways above. (**A**) It indicated the gene regulatory networks from the stage of L4 to D6, including the TFs (NHR-28, UNC-62 and FOS-1 in red triangular frame) as well as the target genes (in pink circle). (**B**) It indicated the gene regulatory networks from the stage of D6 to D15, including the TFs (NHR-28, UNC-62 and FOS-1 in red triangular frame) as well as the target genes (in pink circle). The external part represents the common genes between these two periods and the inner part represents the novel targets not appearing in the other period.

There were totally 154 and 158 individual genes in above five truly significant signaling pathways, respectively from L4 to D6 and from D6 to D15. The expression profiles of these genes were shown in Additional file 4. Based on ChIP–seq data analysis to explore the binding site targets of individual factors, 2530, 5974 and 3178 target genes were identified for transcription factors (TFs) of FOS-1, NHR-28 and UNC-62, respectively (Additional file 4). To dissect the association among these regulators or between regulators and target genes in multiple signaling pathways both from L4 to D6 and from D6 to D15, multi-level gene regulatory networks were constructed for these two developmental stages. As a result, NHR-28 was commonly identified at the top of the network, which regulated UNC-62 and FOS-1, the regulator of FOS-1 was regulated by UNC-62 at the same time (Figure 8). HOX co-factor UNC-62 (Homothorax) has been identified as a developmental regulator that binds proximal to age-regulated genes and modulates lifespan by integrating RNAi and genomics approach [39]. *C. elegans* FOS-1 has been shown to act in uterine and vulval development, which regulates *plc-1* expression in the spermatheca to control ovulation [4]. Moreover, 64 interactions between regulatory factors and target genes were identified at the first stage, including 26 genes regulated by NHR-28, 21 genes by UNC-62 and 17 genes by FOS-1. By contrast, 79 interactions between regulatory factors and target genes were identified at the second stage, including 31 genes regulated by NHR-28, 27 genes by UNC-62 and 21 genes by FOS-1 (Additional file 5). 32 common target genes were identified at both stages, but 3 genes only appears at the first stage (*ruvb-1*, *let-60* and *siah-1* in Figure 8A) and 10 genes only appears at the second stage (*cdc-42*, *kin-19*, *mom-5*,*ife-2*, *mek-2*, *C07E3.9*, *gpa-12*, *sos-1*, *let-23*and *skr-21* in Figure 8B).

In this study, we employed the gene expression datasets of three time points during aging in *C. elegans* and performed the approach of GSEA on each dataset between two adjacent stages. As a result, multiple genetic pathways and targets were identified as significantly down- or up-regulated. Among them, 5 truly aging-dependent signaling pathways including MAPK signaling pathway, mTOR signaling pathway, Wnt signaling pathway, TGF-beta signaling pathway and ErbB signaling pathway as well as 12 significantly associated genes were identified with dynamic expression pattern during aging. On the other hand, the continued declines in the regulation of several metabolic pathways have been demonstrated to display age-related changes. Furthermore, the reconstructed regulatory networks based on three of aging related ChIP–seq datasets and the expression matrices of 154 involved genes in above signaling pathways provided new insights into aging at the multiple pathways level. In conclusion, the identification of these genetic pathways and target genes by our integrated analysis would support the hyperfunction theory, which is a plausible alternative to the molecular damage theory to explain aging in *C. elegans* [1]. The combination of multiple genetic pathways and targets needs to taken into consideration in future studies of aging, in which the dynamic regulation would be uncovered.

## METHODS

### Gene expression data collection and pre-processing

The gene expression dataset of GSE21784 related to aging was collected from the database of GEO (www.ncbi.nlm.nih.gov/geo/), which was contributed by Youngman MJ et al. [14]. In this study, RNA samples were obtained from wild type *C. elegans* strain Bristol N2 during aging and then prepared for hybridization to Affymetrix *C. elegans* Genome Array (GPL200) from synchronized populations of *C. elegans* at three points: the late larval L4 stage (L4, 1 day pre-adulthood), day 6 of adulthood (D6), and day 15 of adulthood (D15). There are three biological replicates for each time point: 3 for L4 (marked with L4-1, L4-2 and L4-3 in the following analysis), 3 for D6 (D6-1, D6-1 and D6-3), as well as 3 for D15 (D15-1, D15-1 and D15-3). We then divided the datasets into two subsets, including the study of period from L4 to D6 (containing 6 samples: L4-1, L4-2, L4-3, D6-1, D6-1 and D6-3) and the study of period from D6 to D15 (also containing 6 samples: D6-1, D6-1, D6-3, D15-1, D15-1 and D15-3). It aims to uncover the diverse mechanism of aging process.

To assess the influence of preprocessing on the comparison, data preprocessing was performed using software packages developed in version2.6.0 of Bioconductor and R version 2.10.1. Each Affymetrix dataset was background adjusted, normalized and log2 probe-set intensities calculated using the Robust Multichip Averaging (RMA) algorithm in Affy package [41].

### The analysis of significantly enriched pathways and genes

The approaches of Gene Set Enrichment Analysis (GSEA) were performed using Category package in version 2.6.0 of Bioconductor to identify the significantly enriched pathways and genes in both above two subsets [42, 43]. In our analysis, the gene sets with less than 10 genes were excluded. The t-statistic mean of the genes was computed in each KEGG (Kyoto Encyclopedia of Genes and Genomes) pathway. Using a permutation test with 1000 times, the cutoff of significance level p value was set as 0.01 for the most significant pathways related to aging. Accordingly, the significant pathways and genes were then identified under the comparison between different time points, including from L4 to D6 and from D6 to D15. The following classification of identified pathways was based on the KEGG pathway maps br08901 of BRITE Functional Hierarchies in KEGG database (http://www.genome.jp/kegg-bin/get_htext?br08901.keg). The annotation of significant C. elegans genes in each pathway was based on the WormBase database, of version WS236 (www.wormbase.org). Next, clustering on groups and genes was performed based on the identified genes' expression in each significant pathway using the method of hierarchical clustering with Euclidean distance. The significance of associated target genes from L4 to D6 or from D6 to D15 was calculated by unpaired t test.

### The collection and analysis of aging related ChIP–seq data

In the database of modENCODE, there are almost one hundred available ChIP–seq data associated with individual transcription factors at diverse developmental stages. Recently, Van Nostrand et al. have performed integrative analysis of these data sets to infer gene regulatory interactions [44]. As a result, nine candidate aging regulators (NHR-28, PQM-1, FOS-1, NHR-76, NHR-77, ELT-3, C01B12.2, UNC-62 and SKN-1) were identified, of which three were previously known to have a role in longevity (ELT-3, SKN-1 and UNC-62). However, there are only 3 datasets at the similar stage (at or after L4 stage) of Matthew JY et al.'s gene expression study, including NHR-28 and FOS-1 at L4 stage (L4) as well as UNC-62 at the stage of young adult day 4 (YD). Therefore, these three ChIP–seq data were then downloaded and analyzed to explore the binding site targets of individual factors. Raw sequencing reads were aligned against C. elegans genome (WS232) using bowtie2 [45]. MACS2 was used for peak calling with default parameters [46].

### Construction of regulatory networks of signaling pathway genes

The tool of Constructing Multi-level Gene Regulatory Networks (CMGRN) was used to integrate NHR-28 (L4), FOS-1 (L4) and UNC-62 (YD) ChIP-seq information of transcription factor (TF) binding with expression profiling of signaling pathway genes that significantly enriched during aging. CMGRN(http://bioinfo.icts.hkbu.edu.hk/cmgrn/) is based on a methodology combining Bayesian hierarchical model with Gibbs Sampling implementation to identify correct sets of regulator-gene interactions which explore underlying network structures [47]. We aimed to dissect the association between these regulators or between regulators and genes as well as to reconstruct regulatory networks in signaling pathways involved in aging processes from L4 to D6 and from D6 to D15. Firstly, the file of sequence counts of our selected 3 regulators (NHR-28, FOS-1 and UNC-62) on the target genes was used here as the input of ChIP–seq gene counts. Secondly, the target gene symbols containing sequences of each regulator measured by ChIP–seq was used as the input of binding data. The last input file is the gene expression profile. Here, we used the gene expression values of involved genes in the significantly identified pathways by GSEA of each sample, respectively for the stages from L4 to D6 and from D6 to D15. Then the parameters were set as defaults: the minimal value of interaction was selected as 10, the probability cutoff for regulator-gene interaction was selected as 70% and the causal relationship percentage was selected as 20%.

## SUPPLEMENTAL DATA


