# Supplementary material for: Dynamic regulation of genetic pathways and targets during aging in Caenorhabditis elegans
Source: Aging (Albany NY). 2014 Mar 31;6(3):215–30. doi: 10.18632/aging.100648 (PMC4012938; doi:10.18632/aging.100648)
Supplement: Supplementary file 1 [file aging-06-215-s001.pdf]

39. Van Nostrand EL, Sanchez-Blanco A, Wu B, Nguyen A and Kim SK. Roles of the developmental regulator unc-62/Homothorax in limiting longevity in Caenorhabditis elegans. PLoS Genet. 2013; 9:e1003325.

40. Hiatt SM, Duren HM, Shyu YJ, Ellis RE, Hisamoto N, Matsumoto K, Kariya K, Kerppola TK and Hu CD. Caenorhabditis elegans FOS-1 and JUN-1 regulate plc-1 expression in the spermatheca to control ovulation. Molecular biology of the cell. 2009; 20:3888-3895.

41. Gautier L, Cope L, Bolstad BM and Irizarry RA. affy--analysis of Affymetrix GeneChip data at the probe level. Bioinformatics. 2004; 20:307-315.

42. Chiaretti S, Li X, Gentleman R, Vitale A, Vignetti M, Mandelli F, Ritz J and Foa R. Gene expression profile of adult T-cell acute lymphocytic leukemia identifies distinct subsets of patients with different response to therapy and survival. Blood. 2004; 103:2771-2778.

43. He K, Chen Z, Ma Y and Pan Y. Identification of high-copper-responsive target pathways in Atp7b knockout mouse liver by GSEA on microarray data sets. Mamm Genome. 2011; 22:703-713.

44. Van Nostrand EL and Kim SK. Integrative analysis of C. elegans modENCODE ChIP-seq data sets to infer gene regulatory interactions. Genome Res. 2013.

45. Langmead B and Salzberg SL. Fast gapped-read alignment with Bowtie 2. Nature methods. 2012; 9:357-359.

46. Zhang Y, Liu T, Meyer CA, Eeckhoute J, Johnson DS, Bernstein BE, Nusbaum C, Myers RM, Brown M, Li W and Liu XS. Model-based analysis of ChIP-Seq (MACS). Genome biology. 2008; 9:R137.

47. Guan D, Shao J, Deng Y, Wang P, Zhao Z, Liang Y, Wang J and Yan B. CMGRN: a web server for constructing multilevel gene regulatory networks using ChIP-seq and gene expression data. Bioinformatics. 2014; Epub ahead of print.

SUPPLEMENTAL DATA

Please browse the full text version of this manuscript to see the Additional files.
